# Iridoschisis: visual outcome in treated versus untreated eye

**DOI:** 10.3205/oc000166

**Published:** 2020-08-26

**Authors:** Eric Ruff, Nataliya Pokeza, Inci Dersu

**Affiliations:** 1 SUNY Albany Medical Center, Albany, New York, United States; 2 Eye Center of Southern Connecticut, Hamden, Connecticut, United States; 3 SUNY Downstate Medical Center, Brooklyn, New York, United States

**Keywords:** iridoschisis, angle closure glaucoma, cataract surgery

## Abstract

A 67-year-old man was referred for iris color change. He was noted to have narrow angles with atrophic iris appearance and visually significant cataracts. The iris findings were consistent with iridoschisis. The patient was recommended to have cataract surgery. Unfortunately, he was lost to follow-up. One year later, he presented with chronic angle closure glaucoma on the right eye with very high pressure and very poor remaining vision. Left-eye vision was also compromised with cataract. Despite the presence of small pupil, abnormal iris stroma, and dense cataract, the patient underwent successful cataract surgery and achieved 20/20 vision post-operatively. Iridoschisis can cause substantial ocular morbidity if not treated timely.

## Introduction

Iridoschisis is a term coined by Loewenstein and Foster in 1945 to describe a rare condition involving separation of iris stroma layers due to iris atrophy after the very first case report by Schmitt [1]. Iridoschisis can compromise the cornea, lens, and optic nerve, and cause blindness [2]. Despite iris atrophy and disintegration and sometimes completely freed up iris pieces, the pupil is usually round and unaffected. Intraocular pressure and the iridocorneal angle could be unremarkable [2]. This can make predicting potential problems such as angle closure glaucoma and corneal decompression difficult. Here, we report a case with iridoschisis and cataracts who developed angle closure glaucoma rather rapidly.

## Case description

**Figure 1:**
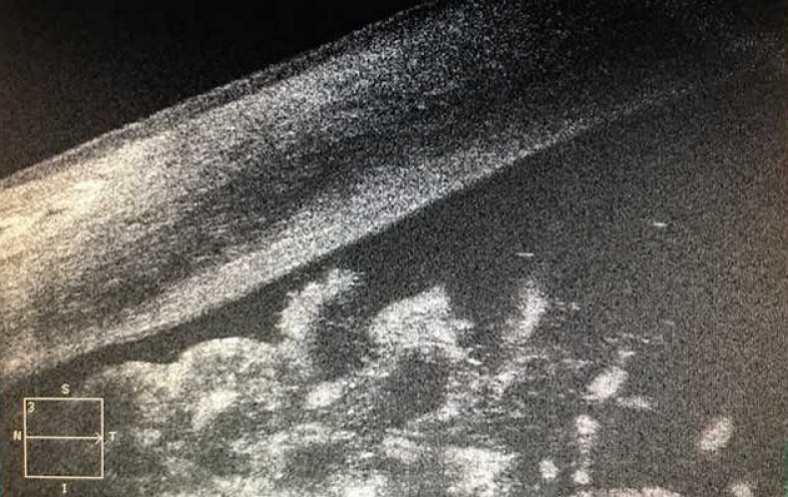
Frayed iris segments blocking the angle view (anterior segment scan, Cirrus HD-OCT, Carl Zeiss Meditec, Dublin, California, USA)

**Figure 2:**
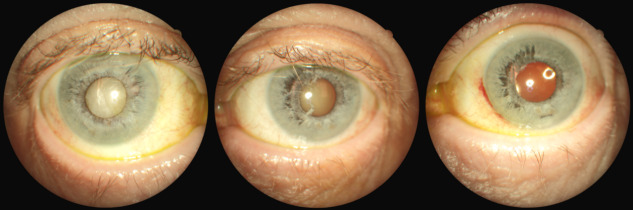
(Left) iridoschisis and cataract right eye; (center) left eye before cataract surgery; (right) after cataract surgery (Canon CR-2 AF Digital Non-Mydriatic Camera, Canon USA, Inc., Lake Success, New York, USA)

We describe a 67-year-old man who was referred from the oncology department due to iris changes which were noted while he was being worked up for metastatic melanoma with unknown source. The patient’s last eye exam had almost been 10 years earlier, and there was no documentation of iris abnormality. The patient himself did not notice if anything was wrong with the eye. His past medical history was also significant for skin basal cell carcinoma. His past social history revealed alcohol and cocaine use. The infection work-up including syphilis was negative. On exam, vision was 20/60 in the right eye and 20/50 in the left eye. Loose iris strands were noted on both eyes, predominantly the left eye. Bilateral cataracts were noticed with narrowing of the angles. Intraocular pressures were normal. On gonioscopic exam, the angle was obscured by iris strands in one quadrant, the rest of the angle seemed narrow. This was confirmed by anterior segment OCT (Zeiss Meditech) (Figure 1 (Fig. 1)). The patient was offered to have cataract surgery in his worse seeing eye first. However, he missed multiple appointments, and when he presented one year later, his vision was reduced to bare light perception in the right eye and 20/150 in the left eye, with IOP measuring 45 mm Hg in the right eye and 12 mm Hg in the left eye. The angle was completely closed in the right eye with a large mature cataract. This time, further iris splitting was noted in addition to visible iris vessels on the right (Figure 2 (Fig. 2)). Advanced nuclear sclerosis and posterior subcapsular cataract were noted on the left eye as well. This time, the patient agreed to cataract surgery on the left eye only. Axial length was shorter than normal, 22.41 mm and 22.49 mm, right and left eye, respectively. The patient underwent successful cataract surgery on the left eye. Vision improved to 20/20 within one month of surgery. The iris strands obtained during the cataract surgery were sent for a pathologic exam. No atypia, inflammation, or reactive changes were found in the specimen.

## Discussion

Iridoschisis is a rare but potentially blinding ocular disease in which the anterior layers of the iris can split off and block the angle or float freely in the anterior chamber [3]. Danias et al. suggested this disease has a genetic predisposition based on observing a mother-daughter case of iridoschisis [4]. It has been associated with various corneal pathology including syphilitic interstitial keratitis, microphtalmos, and nanophthalmos [5]. It can present with hyphema according to a recent report [6]. Differential diagnoses include Axenfeld-Rieger syndrome, iridocorneal endothelial syndrome, and – more commonly – acute angle closure glaucoma. Angle closure glaucoma coexists in half of the iridoschisis cases [1]. Forward bowing of the iris stroma shown by gonioscopy and ultrasound biomicroscopy as well as blockage of trabecular meshwork with free-floating iris pieces have been suggested for the mechanism of the elevated IOP. Salmon and Murray, in a study of a case series of 12 patients, had postulated that multiple subacute angle closure glaucoma attacks may have caused increased IOP and iris atrophy in their patients [7]. They also pointed out similarities between angle closure glaucoma and iridoschisis for both having shorter axial length and shallow anterior chamber [7]. Our patient indeed had short axial length and anterior chamber depth. Interestingly, our patient had an acute angle closure attack in the eye with less iridoschitic changes first, which emphasizes the important role of the phacomorphic glaucoma component in addition to iris changes in this disease. Cataract surgery may be challenging due to a number of reasons. Firstly, the pupil may not dilate well because of an abnormal iris pattern as well as co-existing posterior synechia. Pupil expanding devices including a Malyugin ring can be utilized to overcome this problem [8]. Secondly, these eyes tend to be smaller, and increased IOP and intraoperative posterior pressure may be encountered. The availability of intravenous acetazolamide or mannitol during surgery is important. Finally, loose iris strands can easily block the phaco tip and cause further iris damage. Cutting off the long free iris strands with Vannas scissors eases this problem.

## Conclusion

Iridoschisis is a rare disease with potentially blinding outcome. It is recommended that patients with iridoschisis undergo close observation due to the risk of complications, including acute angle closure glaucoma and blindness. As many eyes may be angle closure suspects regardless of iris splitting, laser iridotomy may be considered in early cases. However, cataract removal ultimately not only helps to open the angle, but also to improve the vision and eye pressure.

## Notes

### Competing interests

The authors declare that they have no competing interests.
